# From Forest to Zoo: Great Ape Behavior Recognition with ChimpBehave

**DOI:** 10.1007/s11263-025-02484-6

**Published:** 2025-06-23

**Authors:** Michael Fuchs, Emilie Genty, Adrian Bangerter, Klaus Zuberbühler, Jean-Marc Odobez, Paul Cotofrei

**Affiliations:** 1 Institute of Information Management, University of Neuchâtel, Neuchâtel, Switzerland; 2 Institute of Biology, University of Neuchâtel, Neuchâtel, Switzerland; 3 Institute of Work and Organizational Psychology, University of Neuchâtel, Neuchâtel, Switzerland; 4 Institute of Behavioural and Neural Sciences, University of St Andrews, St Andrews, Scotland; 5 Perception and Activity Understanding Group, Idiap Research Institute, Martigny, Switzerland; 6 Laboratory of the Idiap Research Institute, EPFL, Martigny, Switzerland

**Keywords:** Non-human primates, Great apes, Chimpanzees, Animal behavior recognition, Video foundation models, Skeleton-based action recognition, Pose estimation

## Abstract

This paper addresses the significant challenge of recognizing behaviors in non-human primates, specifically focusing on chimpanzees. Automated behavior recognition is crucial for both conservation efforts and the advancement of behavioral research. However, it is often hindered by the labor-intensive process of manual video annotation. Despite the availability of large-scale animal behavior datasets, effectively applying machine learning models across varied environmental settings remains a critical challenge due to the variability in data collection contexts and the specificity of annotations. In this paper, we introduce *ChimpBehave*, a novel dataset comprising over 2 h and 20 min of video (approximately 215,000 frames) of zoo-housed chimpanzees, annotated with bounding boxes and fine-grained locomotive behavior labels. Uniquely, *ChimpBehave* aligns its behavior classes with those in PanAf, an existing dataset collected in distinct visual environments, enabling the study of cross-dataset generalization - where models are trained on one dataset and tested on another with differing data distributions. We benchmark *ChimpBehave* using state-of-the-art video-based and skeleton-based action recognition models, establishing performance baselines for both within-dataset and cross-dataset evaluations. Our results highlight the strengths and limitations of different model architectures, providing insights into the application of automated behavior recognition across diverse visual settings. The dataset, models, and code can be accessed at: https://github.com/MitchFuchs/ChimpBehave

## Introduction

The development of machine learning tools to recognize animal behaviors from videos plays a critical role in ecology and ethology. Automated systems for recognizing chimpanzee behaviors could offer a broad spectrum of applications, ranging from enhancing conservation efforts to providing valuable insights into the behavior of great apes. Moreover, non-invasive technologies developed for their well-being can significantly benefit chimpanzees - an endangered species - in both wild and captive settings. For instance, these systems could monitor population dynamics in natural habitats or provide timely signals of behavioral abnormalities in unwell individuals to caretakers in zoos.

**Fig. 1:**
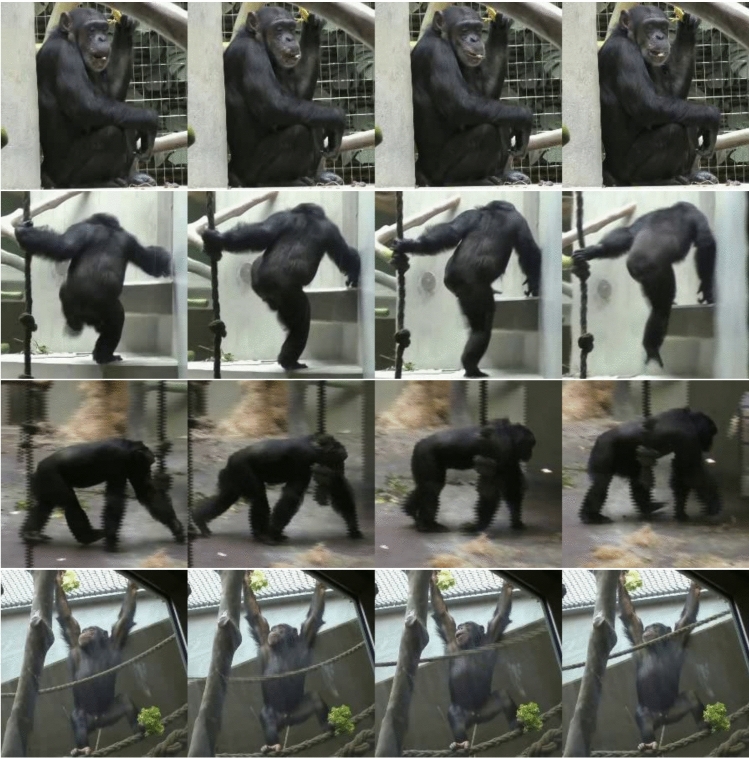
Walking, hanging, sitting, or climbing up? Identifying which chimpanzee behavior is depicted in these images is trivial for most humans. For algorithms, however, this is not always the case, especially when exposed to videos from previously unseen environments. TL;DR: We propose a new dataset and methods to investigate the classification of chimpanzee behaviors across different visual settings

As one of humans’ closest living relatives, chimpanzees have been the subject of extensive scientific research in fields such as ecology, comparative cognition, neuroscience, and evolutionary biology. This research often relies on videos, whose manual annotation is time-consuming and labor-intensive. The advancement of algorithms for animal behavior classification can, therefore, significantly benefit researchers by expediting the labeling process and/or reducing its overall cost.

However, such algorithms require large amounts of data for training before they can be effectively deployed in the observational fields of behavioral studies. To address this, large-scale animal datasets have recently been created to adapt human-centered action recognition models for animal behavior classification (see, e.g., Animal Kingdom (Ng et al. 2022) and MammalNet (Chen et al. 2023)). Although comprehensive, these datasets lack the fine-grained annotations needed to capture the complex behaviors of great apes.

To bridge this gap, more specialized datasets, such as ChimpACT (Ma et al. 2023) and PanAf (Brookes et al. 2024), have been developed to target species-specific behaviors across various environments - ranging from zoo settings to wild forests - and under diverse recording conditions (see Section 2.1 for details). While invaluable, their practical relevance for researchers may be constrained by the distinctiveness of their visual, environmental, and recording settings. For instance, behavior recognition models trained on zoo-specific data may overfit to that context, limiting their ability to generalize to other zoo environments, let alone to data from natural forest habitats. As a result, researchers collecting new data in their own settings may find such models unsuitable unless substantial effort is invested in annotating and fine-tuning them for their specific needs.

A critical factor in animal behavior recognition, therefore, is the model’s capacity to generalize across diverse environments. However, the combined use of ChimpACT and PanAf for cross-dataset analyses is limited by two major factors: (i) each dataset is designed for a distinct computer vision task - spatio-temporal multi-label behavior detection versus multi-class/multi-label behavior recognition; and (ii) their annotated behavior sets are unique and largely unaligned (e.g., ‘resting’ versus ‘sitting’ or ‘standing’). To address these limitations, we introduce *ChimpBehave*, a novel dataset for the recognition of multi-class great ape behavior. ChimpBehave features class labels similar to those of PanAf but is filmed under starkly different visual and recording conditions. It contains footage of chimpanzees in a zoo environment, recorded with handheld cameras focused on individual animals, whereas PanAf comprises footage from stationary outdoor camera traps in natural forests, capturing both chimpanzees and gorillas. This design makes ChimpBehave an ideal resource, either as a stand-alone dataset for great ape behavior recognition or in combination with PanAf, to study cross-dataset generalization.

**Fig. 2:**
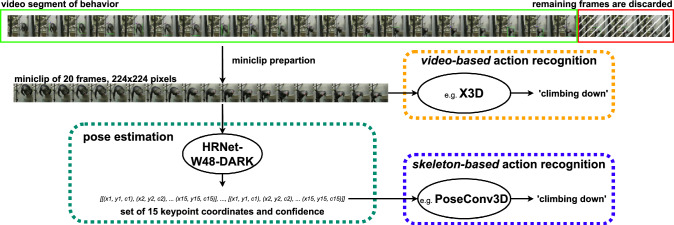
Representation of experiment setting. Miniclips of 20 consecutive frames are extracted from all video segments and fed into VideoMAEv2 or X3D, two video-based action recognition models for behavior classification. In parallel, the individual’s poses are first extracted with HRNet, and then fed into either PoseConv3D, STGCN$++$ or 2 S-AGCN for skeleton-based action recognition

Challenges in cross-domain generalization have been widely explored in human studies. In the past, a promising approach to reduce dependency on domain-specific behavioral data and annotations in humans has been the adoption of *skeleton-based* methods. Unlike *video-based* approaches, which rely on full RGB videos for action inference, skeleton-based methods first extract sequences of skeletal poses as an intermediate representation from which behaviors are predicted. Human-centric studies have shown that such an approach can effectively be used to recognize even fine-grained, complex actions, such as athletes’ movements in gymnastics competitions (Duan et al., 2022; Shao et al., 2020). Furthermore, as highlighted in various reviews on human data (Chen et al., 2017; Feng et al., 2022; Xin et al., 2023), skeleton-based methods tend to be more robust to variations in visual settings, including changes in illumination and background. This robustness is thus particularly advantageous in cross-domain generalization, where models are applied to new environments with differing visual characteristics. Additionally, the reduction in data from high-dimensional video frames to lightweight 2D joint coordinates offers significant benefits, especially in resource-constrained environments, such as low-power field settings (Fuchs et al., 2024).

These promising prospects have attracted the attention of the animal research community, with a growing focus on *pose estimation* - the task of predicting an individual’s posture via joint coordinates. However, translating skeletal poses into practical animal behavior classification has been only marginally explored, particularly in non-laboratory environments.

Indeed, two major challenges arise when considering a skeleton-based approach rather than a video-based one. First, relying solely on skeletal poses while discarding the remaining pixel information could result in lower behavior classification accuracy. Second, accurately estimating the pose of animals presents unique challenges. For example, detecting joint coordinates in animals like chimpanzees is significantly more difficult than in humans due to factors such as the scarcity of publicly annotated data, the variety of poses, frequent occlusions during social interactions, and the low visual contrast of their appearance.

In contrast, a more recent practice in computer vision has been to leverage foundation models pretrained on vast datasets containing billions of data points. These models serve as backbones due to their ability to capture relevant features across a wide range of domains (e.g., ViT-g (Zhai et al. 2022), SwinTransformerv2 (Liu et al. 2022), SAM2 (Ravi et al. 2024), VideoMAEv2 (Wang et al. 2023)). Such an approach could have significant implications for the study of animal behavior, as certain foundation models have demonstrated the ability to generalize well enough to perform comparably to domain-specific models in this area (Sun et al. 2024).

In this context, this paper studies the performance of various video-based and skeleton-based models for great ape behavior recognition, focusing on their generalization capabilities in out-of-distribution settings. Specifically, our experiments target two distinct scenarios:

(i) *Within-dataset* performance, where models are trained and tested on the same dataset, and
(ii) *Cross-dataset* performance, where models are trained on one dataset and tested on a different one.

To thoroughly explore model generalization properties, our study includes a diverse set of model architectures:

- VideoMAEv2 (Wang et al., 2023), a video foundation model and transformer that achieves state-of-the-art (SOTA) performance in several human-centric action recognition benchmarks.
- X3D (Feichtenhofer, 2020), a SOTA video-based 3D Convolutional Neural Network (CNN) architecture that has demonstrated competitive performance with video transformers in great ape behavior recognition in the wild (Brookes et al., 2024).
- PoseConv3D (Duan et al., 2022), a SOTA skeleton-based CNN architecture known for high accuracy in fine-grained human action recognition and proven effective for great ape behavior recognition in the wild (Fuchs et al., 2024).
- STGCN$++$ (Duan et al., 2022) and 2 S-AGCN (Shi et al., 2019), two highly efficient skeleton-based architectures built on Graph Convolutional Networks (GCNs) (Kipf & Welling, 2017), with only 1.4 and 3.5 million parameters, respectively.

In our results section, we present detailed performance metrics for each scenario at both dataset and class levels, employing a rigorous evaluation protocol with stratified 5-fold cross-validation to validate the results and their statistical significance.

In summary, this paper makes the following contributions:

- We introduce *ChimpBehave*, a dataset for great ape behavior recognition, featuring over 2 h and 20 min of video (approximately 215,000 video frames) annotated with fine-grained locomotive behaviors and bounding boxes. Its label classes were annotated by an expert primatologist and specifically aligned with PanAf (Brookes et al., 2024; Sakib & Burghardt, 2021), another dataset designed for the same task but collected under distinct visual and recording conditions. This design enables ChimpBehave to serve as a stand-alone dataset for behavior recognition or, when combined with PanAf, to facilitate cross-dataset generalization experiments.
- We establish a *performance baseline* on ChimpBehave using video-based transformers, 3D-CNNs, and three different skeleton-based models for action recognition.
- We study both *within-dataset* and *cross-dataset generalization* for video-based and skeleton-based methods on ChimpBehave, PanAf, and their combination. Our results show that VideoMAEv2, the large video foundation model, outperforms all other approaches in within-dataset evaluations and demonstrates strong generalization performance in cross-dataset settings. X3D, the other video-based model, ranks second within-dataset but suffers significant performance declines in cross-dataset experiments. In contrast, all skeleton-based models exhibit lower performance within datasets but demonstrate greater robustness to visual changes in cross-dataset scenarios, occasionally even surpassing VideoMAEv2.

This paper is organized as follows. In Section 2, we review related work, introducing various non-human primate datasets used in computer vision applications and providing an overview of key action recognition studies focusing on non-human primates. In Section 3, we present the proposed ChimpBehave dataset, detailing its description, data collection process, and annotation methodology. Section 4 outlines our methods and experiments, including descriptions of the datasets used, the evaluation metrics, and the methodology behind both our video-based and skeleton-based approaches. This section also covers the models, experimental setup, and protocol details. Experimental results are presented in Section 5, while a discussion of our findings, of some limitations, and of suggestions for future research is presented in Section 6.

## Related work

### Non-human primate datasets

The growing number of animal datasets designed for computer vision tasks has prominently included non-human primates (NHP), reflecting their importance across various ethological and ecological studies. These datasets span a wide array, from those encompassing multiple animal orders (Chen et al., 2023; Liu et al., 2023; Ng et al., 2022; Yang et al., 2023) to those focusing specifically on primates (Yao et al., 2023), apes (Brookes et al., 2024; Desai et al., 2022; Ma et al., 2023), monkeys (Sun et al., 2022), and particularly macaques (Bala et al., 2020; Labuguen et al., 2021; Martini et al., 2024).

These datasets showcase notable diversity in annotations and tasks, including species identification (Brookes et al., 2024; Chen et al., 2023; Desai et al., 2022; Yang et al., 2023; Yao et al., 2023), animal detection and tracking (Brookes et al., 2024; Liu et al., 2023; Ma et al., 2023; Yang et al., 2023), pose estimation (Bala et al., 2020; Desai et al., 2022; Labuguen et al., 2021; Liu et al., 2023; Ma et al., 2023; Martini et al., 2024; Ng et al., 2022; Sun et al., 2022; Yang et al., 2023; Yao et al., 2023), and behavior recognition (Bala et al., 2020; Brookes et al., 2024; Chen et al., 2023; Liu et al., 2023; Ma et al., 2023; Martini et al., 2024; Ng et al., 2022).

Among these datasets, only two are dedicated specifically to great ape behavior recognition: ChimpACT (Ma et al., 2023) and PanAf (Brookes et al., 2024). These datasets adopt distinct approaches, highlighting the challenges of applying computer vision across diverse contexts. ChimpACT captures the daily life of a young chimpanzee in a zoo environment, characterized by man-made backgrounds, dynamic handheld camera movements, and a longitudinal focus on a single individual. In contrast, PanAf documents the behaviors of chimpanzees and gorillas in their natural habitats, using static cameras deployed in African forests to capture a wide variety of ape populations. These differing settings underline the challenges faced by computer vision models in adapting to varying visual environments. Table 1 provides a comparative overview of the main features of these datasets, while Table A5 in the Supplementary Materials compares their annotated locomotion behavior classes.

**Table 1:** Main feature comparison of ChimpBehave, PanAf and ChimpACT for Behavior Recognition (BR) and Detection (BD)

	ChimpBehave	PanAf		ChimpACT
		P500	P20K	
Species	Chimpanzees	Chimpanzees & Gorillas		Chimpanzees
Environment	Zoo / Man-made	Forest / Natural		Zoo / Man-made
Location	Basel (CH)	Tropical Africa		Leipzig (D)
Recording method	Focal sampling	Camera trapping		Focal sampling
Cameras	Moving	Fix		Moving
Resolution	1920x1080@25	720x404@24		720x578@25 / 1280x720@25
Annotated frames	213,000	180,000	7,000,000	160,000
Annotated by primatologist	✓	✗	✗	✗
Locomotion behaviors	8	7	3	4
Total behaviors	8	9	18	23
Task	Multi-Class BR	Multi-Class BR	Multi-Label BR	Multi-Label BD
Image examples	Fig. A1	Fig. A2		Fig. A3

PanAf is further divided into two subsets: PanAf20K and PanAf500. PanAf20K comprises over 7 million frames labeled with broad ecological behaviors through crowdsourcing, designed for multi-label behavior recognition at the video level. In contrast, PanAf500 is a smaller, fine-grained dataset focused on multi-class behavior recognition, with individual bounding boxes annotated with their corresponding behaviors. In this paper, we focus on PanAf500 for its precise annotation scheme, referring to it as PanAf for simplicity.

While ChimpACT and PanAf are valuable datasets, they exhibit limitations when considered for combined use in primatology research. ChimpACT targets spatio-temporal behavior detection with annotations emphasizing social interactions and broad locomotive behaviors (e.g., ‘moving’, ‘resting’, ‘climbing’). PanAf, however, focuses on fine-grained individual locomotive actions, such as ‘walking,’ ‘running,’ ‘sitting,’ and ‘climbing down.’ These differences in design and annotation complicate cross-dataset analyses, making direct model comparisons challenging.

ChimpBehave bridges this gap by combining a visual and filming setup reminiscent of ChimpACT, with recordings of individuals in man-made environments featuring camera motion and zooming, while aligning its behavioral annotation scheme with PanAf’s detailed locomotive action categories. Its design makes it a valuable resource for primatology, suitable for use as a stand-alone dataset or in combination with PanAf, for experiments in out-of-distribution generalization.

### Behavior Recognition for Non-Human Primates

With advancements in deep learning, including Convolutional Neural Networks (CNNs) and Transformers, several studies have applied these techniques to the automated recognition of NHP behaviors. To date, these efforts have primarily focused on macaques (Bala et al., 2020; Li et al., 2023; Marks et al., 2022), monkeys (Liu et al., 2023), and apes (Bain et al., 2021; Brookes et al., 2023, 2024; Fuchs et al., 2024; Ma et al., 2023; Sakib & Burghardt, 2021). Key advancements in this domain leverage action recognition (AR) techniques, which are essential for classifying behaviors from video sequences. Below, we provide an overview of recent research focusing on great apes. These techniques are generally categorized into three main approaches: video-based, skeleton-based, and multimodal.

**Video-Based AR:** Video-based action recognition analyzes visual features directly from raw video data, capturing movements and interactions within the pixel data of each frame. Several studies have employed such methods specifically for NHP behavior recognition. In Sakib and Burghardt (2021), experiments on the PanAf dataset were conducted using C2D (Simonyan & Zisserman, 2014), an early dual-stream model architecture that fuses RGB and optical flow data before classification. Subsequently, Brookes et al. (2023) expanded the number of data streams by incorporating a third path to include dense pose, as presented in Sanakoyeu et al. (2020). The PanAf dataset was later benchmarked in Brookes et al. (2024) using various CNN-based and Transformer-based models.

Additionally, several models for spatiotemporal action detection - detecting actions in both space and time within videos - have been benchmarked on videos annotated with chimpanzee behaviors in ChimpACT (Ma et al., 2023).

**Skeleton-Based AR:** Skeleton-based approaches, in contrast, focus on tracking the movement of key body points or joints, constructing a skeletal representation of the subject to recognize specific actions or behaviors. This approach was first applied to great ape behavior classification in ASBAR (Fuchs et al., 2024), a framework that combines DeepLabCut pose estimation modules (Lauer et al., 2022; Mathis et al., 2018) with PoseConv3D (Duan et al., 2022) for action recognition.

**Multimodal AR:** The recent rise of Vision-Language Models (VLMs) (Zhang et al., 2024), which combine video frames and text as input, was first adapted for great ape behavior classification in Brookes et al. (2024). In addition, Bain et al. (2021) incorporated multimodal signals by including audio cues to enhance chimpanzee behavior classification.

## The ChimpBehave Dataset

In this section, we present the details of the dataset we have built and its main features.

**Dataset Description:** ChimpBehave consists of 1,362 annotated video segments of chimpanzees, each labeled for multi-class action recognition, specifying a unique behavioral class along with the individual’s corresponding bounding boxes. These segments were derived from a collection of 50 longer videos recorded in 2016 at the Basel Zoo indoor enclosure (see examples in Fig. 1 and Fig. A1 in Supplementary Materials).

At the time of the study, the Basel Zoo housed a group of 15 chimpanzees (*Pan troglodytes verus*), including 12 females (6 adults, 1 subadult, 3 juveniles, and 2 infants) and 3 males (1 adult, 1 juvenile, and 1 infant). The group resides in a facility consisting of six indoor lodges (totaling 233.3 m²) and two outdoor enclosures (totaling 477 m²). The lodges are equipped with climbing structures, ropes, puzzle boxes, and other enrichment features.

Each original video was captured using *focal sampling*, where one of nine chimpanzees was tracked while also capturing its surroundings and other conspecifics. The filming conditions were naturalistic, including camera motion, zooming, and occasional shaking. All videos were recorded with a handheld camera at a resolution of 1920x1080 pixels and 25 fps.

The annotated video segments were selected for their clear depiction of behaviors, with an emphasis on including less frequent locomotive behaviors whenever possible. In total, the dataset comprises approximately 215,000 annotated video frames.

**Fig. 3:**
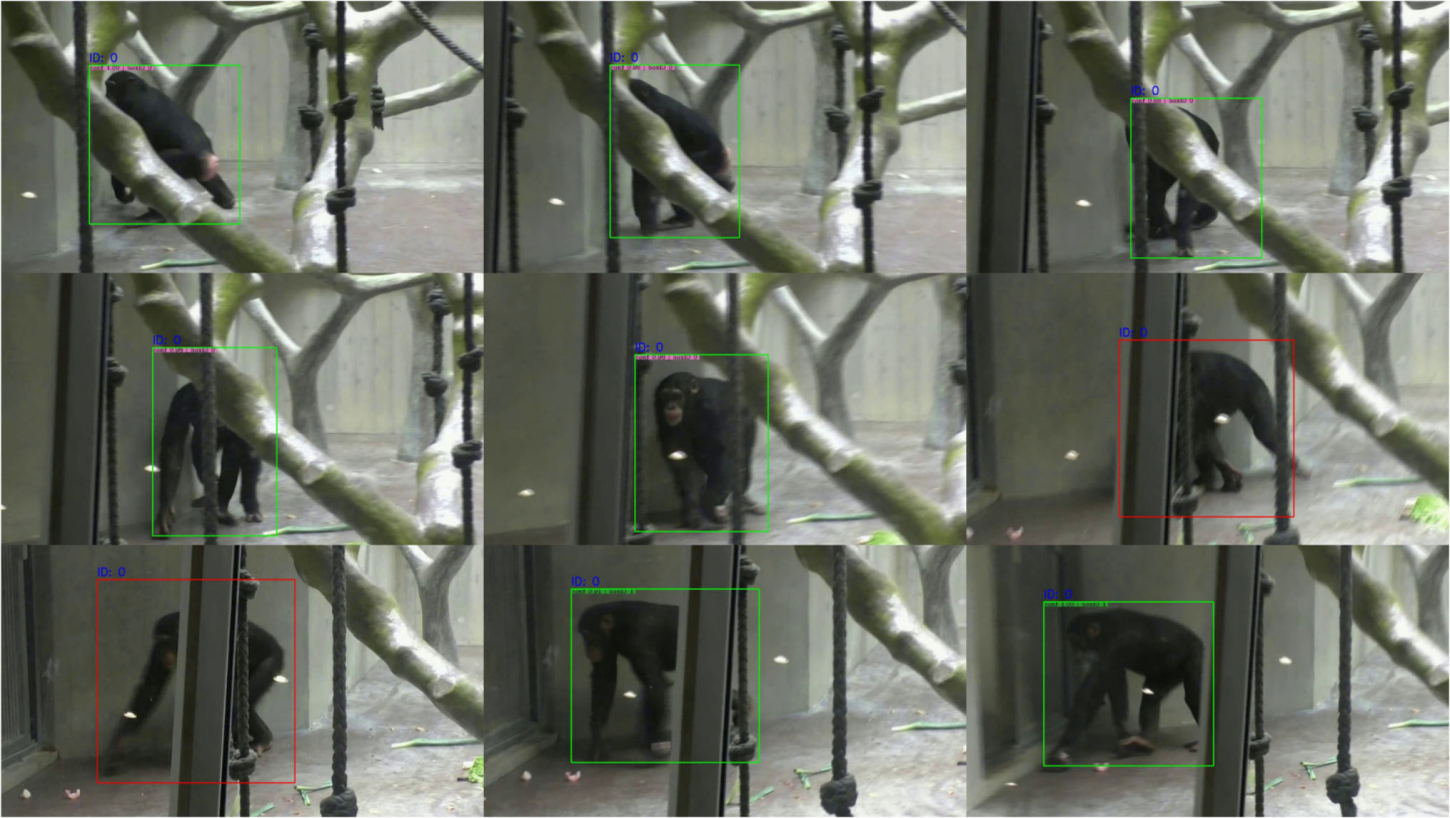
Tracking example after correcting IDs and interpolating missing frames (red bounding box). When the individual passed behind the pole, the network lost its track and assigned a new ID. All predicted tracks were manually reviewed, and proper IDs reassigned programmatically where necessary. Missing frames were reconstructed using linear interpolation. Best seen zoomed in. Code is available in the project repository (Color figure online)

**Fig. 4:**
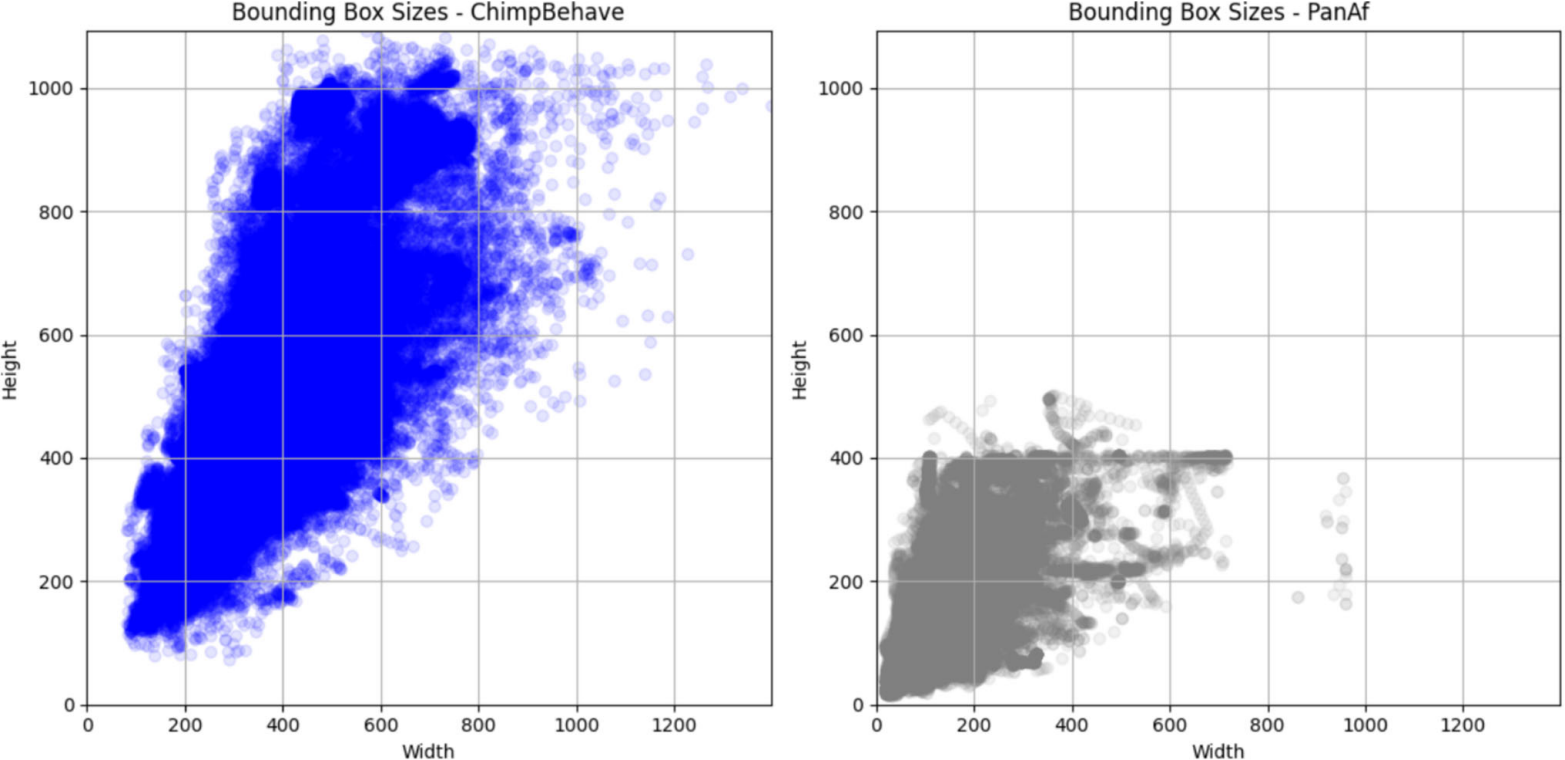
Bounding box sizes in the ChimpBehave (*left in blue*) and PanAf (*right in grey*) datasets. Each dot represents the size of one bounding box annotated with behavior (Color figure online)

**Bounding Box Annotations:** To label all video frames with bounding boxes, we employed a three-stage process, described below:

1. A single annotator (MF) manually labeled over 16,000 video frames from 206 video segments. Annotations were done on every tenth frame, with an interpolation approach used for the remaining frames. All annotations were performed in Label Studio (https://labelstud.io/) across a variety of scenes. For each focal chimpanzee, a minimum of 500 frames were annotated, and for each of the 50 original focal videos, at least two segments or 130 frames were included.
2. These labels were then used to fine-tune QDTrack (Pang et al., 2021) (MMAction2 Contributors, 2020), a state-of-the-art Multiple Object Tracking (MOT) model pretrained on the ChimpACT dataset (Ma et al., 2023). This model was selected due to its demonstrated effectiveness for this task, as highlighted in Ma et al. (2023). Fine-tuning was conducted for 6 epochs using 168 video segments, leaving 38 segments for evaluation. The final model achieved the following scores on common MOT metrics at test time: Recall 0.576, Precision 0.706, HOTA 0.499, mAP 0.557.
3. We then used this model to predict tracking bounding boxes for all video segments, manually refining the tracks to correct any ID swaps and linearly interpolating predictions where necessary (see Fig. 3 for more details). Each track was individually reviewed, and only valid sequences were retained in the final dataset. The distribution of bounding box sizes is visualized in Fig. 4. The code for MOT fine-tuning and data conversion between MMAction2 and Label Studio is available in our code repository. Fig. A13 provides further details on our iterative fine-tuning process.

**Keypoint Annotations:** The coordinates of 1,500 keypoints were manually annotated by MF. This includes 15 keypoints in 100 individual frames, with two frames selected from each of the 50 focal videos.

**Behavior Annotations:** An expert primatologist (EG) meticulously labeled the dataset’s behavior annotations for all video segments using ELAN (https://archive.mpi.nl/tla/elan). The annotations focused on eight mutually exclusive behavioral classes representing common primate locomotor behaviors: ‘sitting,’ ‘standing,’ ‘walking,’ ‘running,’ ‘hanging,’ ‘swinging,’ ‘climbing down,’ and ‘climbing up’ (see full ethogram in Table A4 in the Supplementary Materials). Interrater reliability was deemed unnecessary, as these fundamental and clearly defined behaviors make disagreements among annotators highly unlikely.

These classes were selected because they represent a diverse range of locomotion and posture-related behaviors commonly exhibited by primates. They capture both stationary (e.g., ‘sitting,’ ‘standing’) and dynamic (e.g., ‘walking,’ ‘running,’ ‘swinging’) actions, providing a comprehensive dataset for action recognition.

From an ecological perspective, these behaviors are integral to understanding primate activity patterns, energy expenditure, and environmental interactions. For instance, locomotor behaviors like ‘climbing up’ and ‘swinging’ are directly tied to arboreal navigation, which is critical for species living in forested habitats. Similarly, stationary behaviors such as ‘sitting’ and ‘standing’ are often associated with feeding or resting, offering insights into primates’ social and foraging strategies.

**Fig. 5:**
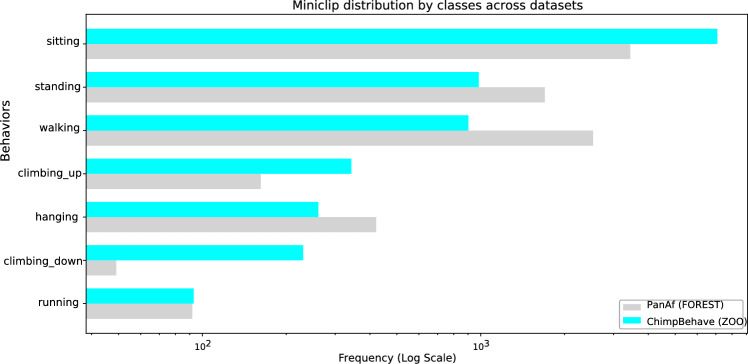
Behavior frequency distribution in the ChimpBehave and PanAf datasets, used in our experiments. Frequencies are plotted on a logarithmic scale to highlight the long-tailed characteristic of the data

In addition, seven of these classes were intentionally selected to match those annotated in Brookes et al. (2024), in order to facilitate cross-dataset analysis. Similar to Brookes et al. (2024), we observe a long-tail class distribution in our dataset, as shown in Fig. 5. Accordingly, we refer to ‘sitting’, ‘standing’, and ‘walking’ as *head* classes, while the remaining five are considered *tail* classes.

## Method and Experiments

Our objective is to study and compare within-dataset and cross-dataset generalization performance of a varied range of video-based and skeleton-based action recognition models. To achieve this, we first describe the datasets and data preparation process, followed by the evaluation metrics used in our experiments. We then describe the video-based behavior recognition model and its implementation details, followed by the skeleton-based recognition model, including keypoint estimation. See Fig. 2 for on overview of our data pipeline. Finally, we outline the experimental protocol.

### Datasets and Data Preparation

In addition to ChimpBehave (described in Sect. 3), we relied on three additional datasets introduced below.

#### OpenApePose

The OpenApePose dataset (Desai et al., 2022) comprises 71,868 images annotated with the 2D poses of various ape species, namely chimpanzees (approximately 25%), gorillas (18%), orangutans (18%), bonobos (16%), gibbons (13%), and siamangs (10%). The images capture individuals in diverse settings, including zoos, sanctuaries, and field sites (see examples in Fig. A4 in the Supplementary Materials). Each image contains pose annotations for one individual, including 17 keypoints: nose, eyes, head, neck, shoulders, elbows, wrists, sacrum (center between hips), knees, and ankles. We used this dataset to train our pose estimation model (Sect. 4.4.1).

#### ChimpACT

ChimpACT (Ma et al., 2023) is a dataset that includes videos annotated for animal tracking, pose estimation, and action detection. It comprises 16,028 images annotated for multi-animal 2D pose estimation, with a total of 56,324 annotated poses from a group of chimpanzees housed at the Leipzig Zoo, Germany. The environment is man-made, and the labeled images are derived from 163 longer video segments (see Section A3 for more details and image examples in Fig. A3 in the Supplementary Materials). It includes 16 labeled keypoints: eyes, upper and lower lips, neck, shoulders, elbows, wrists, root of hips, knees, and ankles. This dataset was also used to train our pose estimation model (Sect. 4.4.1), and we used its tracking data to pretrain our bounding box tracker (Sect. 3).

#### PanAf

The Pan African Programme ‘The Cultured Chimpanzee’ (http://panafrican.eva.mpg.de/index.php) aims to enhance the understanding of evolutionary and ecological factors that influence chimpanzee behavioral diversity. As part of this effort, numerous hours of footage were collected using camera traps placed in the forests of Central Africa. From this collection, 500 videos, each 15 s long (totaling 180,000 frames at 24 fps, resolution 720x404), were annotated with bounding boxes for ape detection and action labels for behavior recognition (Brookes et al., 2024; Sakib & Burghardt, 2021) (see Fig. A2 in the Supplementary Materials for image examples). The nine annotated behaviors include ‘walking’, ‘standing’, ‘sitting’, ‘running’, ‘hanging’, ‘climbing up’, ‘climbing down’, ‘sitting on back’, and ‘camera interaction’. This dataset is used in our experiments for action recognition (Sect. 5). Note that this dataset is referred to as PanAf500 in Brookes et al. (2024), distinct from PanAf20k, which includes coarser-grained actions and is not employed in our experiments.

#### Data Preparation

**Behavioral Class Selection:** In our experiments on ChimpBehave and the results presented in Sect. 5.1, we included all eight behavioral classes in our training and testing data. However, when examining within-dataset and cross-dataset generalization in Sect. 5.2 and Sect. 5.3, we only included the seven classes that overlap with PanAf, namely ‘walking’, ‘standing’, ‘sitting’, ‘running’, ‘hanging’, ‘climbing up’, and ‘climbing down’.

**Miniclip Preparation:** For our experiments, we converted both ChimpBehave and PanAf videos into *miniclips*, i.e., sequences of 20 consecutive video frames associated with one unique behavioral class and without frame overlap. Any remaining frames beyond the last multiple of 20 were discarded. The coordinates of the individual’s bounding boxes within each miniclip were used to calculate the global minimum and maximum coordinates of the region to crop. Each miniclip was then resized to 224x224 pixels. This process yielded 10,043 unique miniclips from ChimpBehave and 8,404 from PanAf.

**Keypoint Extraction:** In our skeleton-based approaches, each miniclip mentioned above was converted into a series of keypoint coordinates and confidence scores using the Dark-HRNet-W48 model, as described in Sect. 4.4.1.

### Evaluation Metrics

We describe below the metrics used in our experiments for pose estimation and behavior recognition. Their mathematical formulas can be found in Section B of the Supplementary Materials.

**Pose Estimation:** To evaluate the performance of our pose estimation model on images from ChimpBehave and PanAf, we relied on the Normalized Mean Error Rate (NMER) and Percentage of Correct Keypoint - Nasal Dorsum (PCK-ND), two metrics suggested in Fuchs et al. (2024), where the set of 2D pose annotations from PanAf were made available.

- *Normalized Mean Error Rate (NMER)* measures the average error distance per image and per keypoint between a predicted keypoint and its ground truth coordinates, normalized by a factor proportional to the dimensions of the individual’s bounding box.
- *Percentage of Correct Keypoint - Nasal Dorsum (PCK-ND)* gives the percentage of keypoints predicted within a certain radius of the ground truth coordinates. In this case, the radius is defined by the length of the individual’s nasal dorsum (nose bridge).

**Behavior Recognition:** At the behavioral class level, we compute the following metrics:

- *Precision*: The proportion of correctly predicted miniclips of a behavioral class among all miniclips predicted as that class.
- *Recall*: The proportion of actual miniclips of a behavioral class that are correctly recognized by the model.
- *F1 Score*: The harmonic mean of Precision and Recall, balancing both metrics, which often involve a trade-off.
- *False Positive Rate (FPR)*: The proportion of miniclips that do not belong to a class but are incorrectly predicted as belonging to it.
- *False Negative Rate (FNR)*: The proportion of actual miniclips of a class that are missed by the model.

At the dataset level, we evaluate each model using the following action recognition metrics: Top-1 Accuracy, F1 Score (weighted), Mean Class Accuracy (MCA), and Mean Average Precision (mAP).

- *Top-1 Accuracy* measures the proportion of correctly classified miniclips, making it sensitive to imbalanced class distributions.
- *F1 Score (weighted)* is useful for imbalanced datasets, as it combines precision and recall, providing a single score while accounting for the distribution of classes.
- *Mean Class Accuracy (MCA)* evaluates the average accuracy across all classes, using the same weight per class.
- *Mean Average Precision (mAP)* provides a comprehensive measure by averaging precision across different recall levels for each class, capturing the model’s overall ability to identify relevant miniclips. This metric is not sensitive to imbalanced class distributions.

### Video-Based Behavior Recognition

**Behavior Recognition Models:** Two models were considered to evaluate the performance of video-based models.

(i) VideoMAEv2 (Wang et al., 2023), a recent video foundation model built on a transformer architecture currently achieving state-of-the-art performances on several human-centric action recognition benchmarks. VideoMAEv2 is notably pretrained in a self-supervised manner over more than a million videos.
(ii) X3D (Feichtenhofer, 2020), a standard action recognition CNN-based model selected due to its strong performance on the PanAf benchmark (Brookes et al., 2024), achieving results comparable to more recent video transformers such as MViTV2 (Li et al., 2022) and TimeSformer (Bertasius et al., 2021). X3D’s architecture incrementally builds upon a small 2D image classification model, expanding along several network dimensions, including space, time, depth, and width. This progressive expansion is designed to optimize the trade-off between model complexity and performance, enabling highly efficient models without sacrificing accuracy.

**Implementation Details:** Each video-based model was trained from weights pretrained on Kinetics 400 (Kay et al., 2017) for 10 epochs using default hyperparameters on 4 GPUs. VideoMAEv2 and X3D use a batch size of 2 and 8, respectively, with clips of 16 consecutive frames. Training was performed using *SGD* optimization, with an initial learning rate of 0.005 and cosine annealing, momentum of 0.9, and weight decay of 0.0001.

### Skeleton-Based Behavior Recognition

The quality of the skeleton-based action recognition model depends heavily on the capacity of the pose estimation model to reliably detect and localize keypoints. In the following section, we first present the design and training of the keypoint detector module, followed by the behavior recognition models.

#### Pose Estimation

**Data Preparation:** We merged images and pose annotations from the OpenApePose and ChimpACT datasets, resulting in a total of 87,896 images annotated with 128,192 poses, comprising chimpanzees (approximately 58%), gorillas (10%), orangutans (10%), bonobos (9%), gibbons (7%), and siamangs (6%). Only keypoints present in both datasets were included: ‘left eye’, ‘right eye’, ‘neck’, ‘left shoulder’, ‘right shoulder’, ‘left elbow’, ‘right elbow’, ‘left wrist’, ‘right wrist’, ‘hip/sacrum’, ‘left knee’, ‘right knee’, ‘left ankle’, and ‘right ankle’. Additionally, we unified the ‘nose’ coordinates from OpenApePose and the ‘upper lip’ coordinates from ChimpACT into a single keypoint labeled ‘nose or upper lip’. This merged dataset was split into train/validation/test partitions using a 68/16/16 split.

**Model Training:** We trained an HRNet-W48 model (Sun et al., 2019), enhanced with the DARK method (Zhang et al., 2020), due to its superior performance on ChimpACT, as demonstrated in Ma et al. (2023). The weights were initialized from a model pretrained on COCO-WholeBody V1.0 (Jin et al., 2020), and fine-tuned for 120 epochs. Additionally, we enforced that each keypoint should be considered *visible*, regardless of whether the original annotation suggested otherwise, to encourage the network to predict joint coordinates even when occluded.

**Fig. 6:**
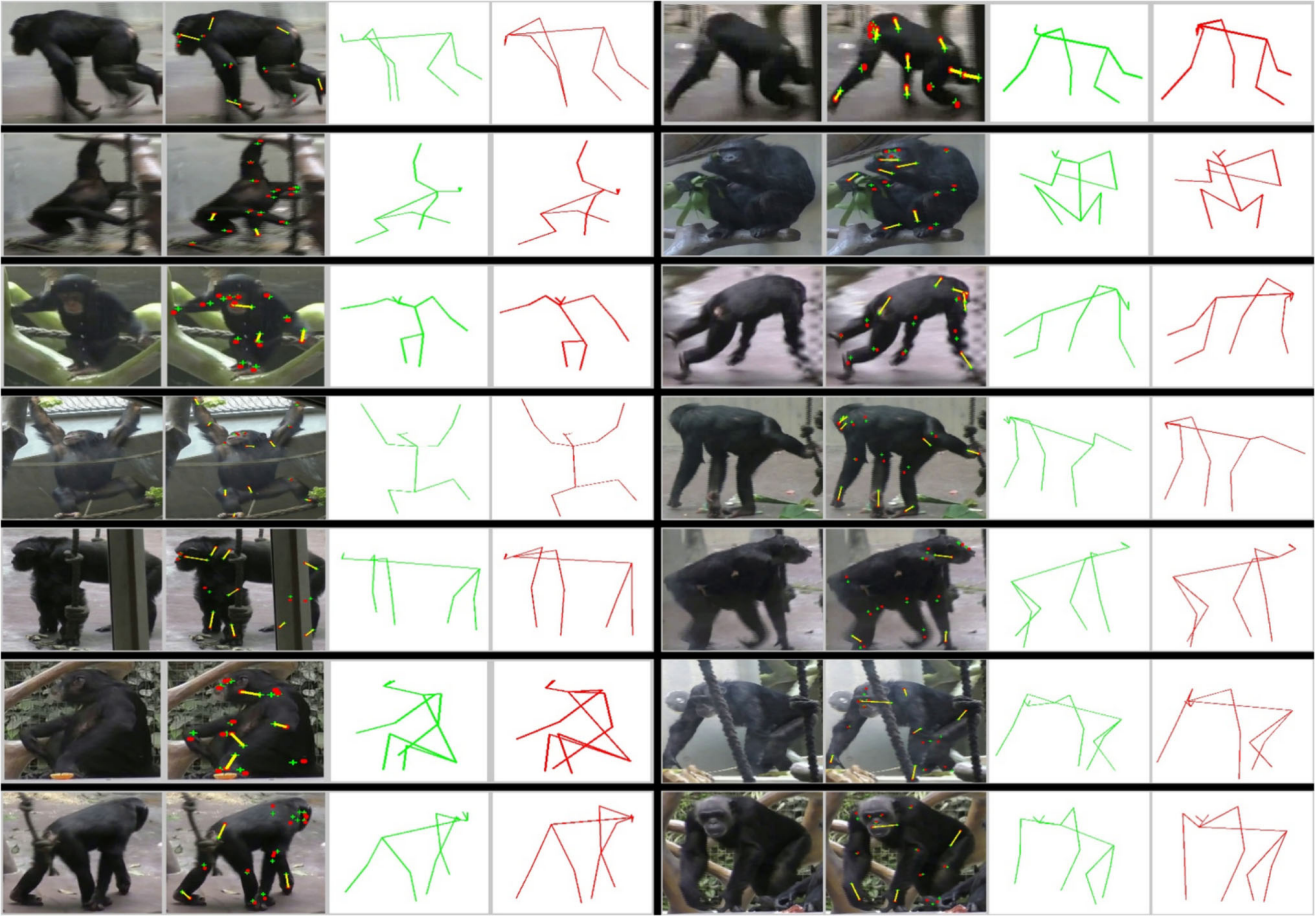
Pose estimation examples on ChimpBehave. From left to right: 1) original image cropped around the individual bounding box. 2) keypoint ground truth (green crosses) and HRNet prediction (red dots), the prediction error is highlighted when greater than the nasal dorsum length (yellow segments). 3) ground truth skeleton (in green). 4) predicted skeleton (in red). Here we present some examples where we consider the pose prediction to be relatively good, ie. where both skeletons share relatively similar appearances. See Figs. A5 to A9 in Supplementary Material for more examples (Color figure online)

**Evaluation on ChimpBehave and PanAf:** To assess the model’s prediction accuracy on ChimpBehave, we annotated 1,500 ground truth keypoint coordinates and used them to compute the Normalized Mean Error Rate (NMER) and PCK-Nasal Dorsum (PCK-ND). We applied the same metrics to images from the PanAf dataset, using keypoint coordinates from Fuchs et al. (2024). Results are shown in Table 2, whereas qualitative prediction examples are shown in Fig. 6 and in Figs. A5 to A8 in the Supplementary Materials.

**Table 2:** Evaluation metrics of our pose estimation network on an image subset of ChimpBehave and PanAf

	# images	# keypoints	NMER	PCK-ND
ChimpBehave	100	1500	9.48%	47.0%
PanAf [in Fuchs et al. (2024)]	320	4800	9.08%	59.19%

**Fig. 7:**
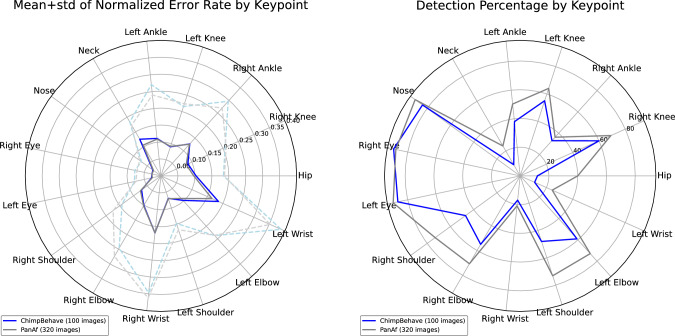
Pose estimation metrics by keypoint for both datasets. (*Left*) Normalized error rate: mean (solid) and mean+std (dotted). (*Right*) Detection percentage within nasal dorsum distance

Several observations can be made based on these results: 1) Despite training on a relatively large dataset, the model achieves only moderate performance on both datasets, failing to correctly detect even half of the keypoints on ChimpBehave (PCK-ND: 47%); 2) Similar to Fuchs et al. (2024), not all keypoints are equally easy to detect. For instance, facial features are detected more accurately than limb extremities (see keypoint-wise metrics in Fig. 7); 3) Despite ChimpBehave’s higher image resolution compared to PanAf, the metrics indicate better pose estimation performance on PanAf. A possible explanation is that PanAf’s pose annotations in Fuchs et al. (2024) were primarily made on video frames depicting less dynamic behaviors, such as ‘walking,’ ‘sitting,’ and ‘standing.’ In contrast, our set of annotations in ChimpBehave deliberately included more challenging poses from fast-paced behaviors, such as ‘climbing down’ and ‘running.’

#### Behavior Recognition Models

Three different skeleton-based models were considered for comparison in our experiments, i. e. STGCNN$++$ (Duan et al., 2022), 2 S-AGCN (Shi et al., 2019) and PoseConv3D (Duan et al., 2022).

(i) On one hand, STGCNN$++$ and 2 S-AGCN are two traditional skeleton-based model architectures relying on Graph Convolutional Networks (GCN) (Kipf & Welling, 2017). Both models transform the individual’s pose coordinates into graphs (or *skeletons*) made of nodes (keypoints) and their connecting edges (limbs), on which convolutions can be applied. Their architectures are therefore extremely lightweight with only 1.4 and 3.5 million parameters, respectively. Both models have shown comparable results on skeleton-based human-centric benchmarks (Duan et al., 2022).
(ii) On the other hand, PoseConv3D uniquely uses CNNs for skeleton-based action recognition, which tends to make it generally more robust in handling noisy pose estimated data (Feng et al., 2022; Yue et al., 2022). Additionally, PoseConv3D has been shown to achieve higher accuracy in recognizing complex human actions (Duan et al., 2022), and was already proven effective for great ape behavior recognition, as shown in Fuchs et al. (2024). The model works by transforming keypoint coordinates and confidence scores into 3D heatmap volumes, which are then processed by a 3D-CNN classifier for action recognition [see Duan et al. (2022) for more details].

**Implementation Details:** All skeleton-based models were trained for 20 epochs using default hyperparameters on 4 GPUs with weights pretrained on NTU60 (Shahroudy et al., 2016) for STGCNN$++$ and 2 S-AGCN, and on FineGym (Shao et al., 2020) for PoseConv3D. Each model was trained using *SGD*, a batch size of 32, an initial learning rate of 0.04, with cosine annealing, momentum of 0.9, and weight decay of 0.0001.

All models use *keypoint* data only, excluding *limb* data, and additionally PoseConv3D did not employ *RGB+Pose multimodality* to clearly differentiate between skeleton-based and video-based approaches.

### Experimental Protocol

**Within-Dataset Cross-Validation Procedure:** To validate all within-dataset experimental results, we followed a standard stratified 5-fold cross-validation procedure. In this approach, each of the five models was trained on 4 folds (representing 80% of the dataset) and validated on the remaining 20%. This ensures that all dataset miniclips are used exactly once for validation. The same folds were used for both video-based and skeleton-based methods. To maintain a similar class distribution across folds, miniclips were sampled proportionally for each class. Miniclip selection for each fold was done in the order of their appearance in the database, sorted by video name and then by frame numbering. This approach groups miniclips at the video level as much as possible, ensuring better generalization across videos, similar to the train/validation/test partitioning used in Brookes et al. (2024).

**Cross-Dataset Cross-Validation Procedure:** In the cross-dataset evaluation, we used all models trained during a cross-validation iteration on one *source* dataset (i.e., using 80% of the miniclips from one dataset) and tested them on all the miniclips of the second *target* dataset. This procedure allows us to assess the generalization capability of the models across datasets.

**Confidence Intervals:** To assess the statistical significance of the results, we calculated 95% confidence intervals based on the Student distribution with degrees of freedom $\nu =4$, for the average of each metric calculated using the five evaluations from the 5-fold cross-validation procedure. Practically, if two confidence intervals calculated for the same evaluation metric but on different models do not overlap, the model with the higher evaluation value can be considered statistically more efficient than the other.

**Experimental Details:** All training and evaluation for the models were conducted using the MMaction2 platform (MMAction2 Contributors, 2020). The experiments were run on the HPC cluster at the University of Neuchâtel, utilizing 4x NVIDIA RTX 2080 Ti GPUs (each with 11GB of memory). For model selection, we chose the final epoch based on its Top-1 Accuracy on the validation set.

## Results

First, in Section 5.1, we present the performances of all models on the ChimpBehave dataset, which includes eight behavioral classes. Subsequently, we incorporate data from the PanAf dataset, focusing on the seven classes common to both datasets, to evaluate and compare within-dataset and cross-dataset generalization for all models in Sections 5.2 and 5.3.

**Table 3:** Mean performance metrics and 95% confidence intervals (CI) for all models on the ChimpBehave dataset with all 8 classes. Acronyms indicate Video-Based (VB) or Skeleton-Based (SB) approaches, Vision Transformers (ViT), Unlabeled Hybrid (UH) - a collection of 1.35M videos [see Wang et al. (2023) for details]

		Model	Mode	Type	Pretraining	Top1	F1	MCA	mAP
ChimpBehave	mean	VideoMAEv2	VB	ViT	UH/K400	**92**.**3**	**92**.**3**	**74**.**8**	**80**.**9**
		X3D	VB	CNN	K400	89.3	89.4	62.8	69.5
		PoseConv3D	SB	CNN	FineGym	86.8	86.3	51.3	57.9
		STGCN$++$	SB	GCN	NTU60	86.7	86.1	49.1	55.0
		2 S-AGCN	SB	GCN	NTU60	86.8	86.4	50.8	56.0
	CI	VideoMAEv2	VB	ViT	UH/K400	[90.1, 94.5]	[90.5, 94.2]	[68.8, 80.8]	[76.1, 85.8]
		X3D	VB	CNN	K400	[86.0, 92.6]	[86.9, 91.9]	[59.4, 66.3]	[62.2, 76.8]
		PoseConv3D	SB	CNN	FineGym	[84.0, 89.6]	[84.4, 88.2]	[48.8, 53.7]	[52.5, 63.4]
		STGCN$++$	SB	GCN	NTU60	[84.9, 88.6]	[84.7, 87.6]	[46.9, 51.3]	[50.7, 59.2]
		2 S-AGCN	SB	GCN	NTU60	[84.7, 89.0]	[85.1, 87.7]	[47.5, 54.2]	[50.3, 61.8]

### Behavior Recognition: ChimpBehave

**Fig. 8:**
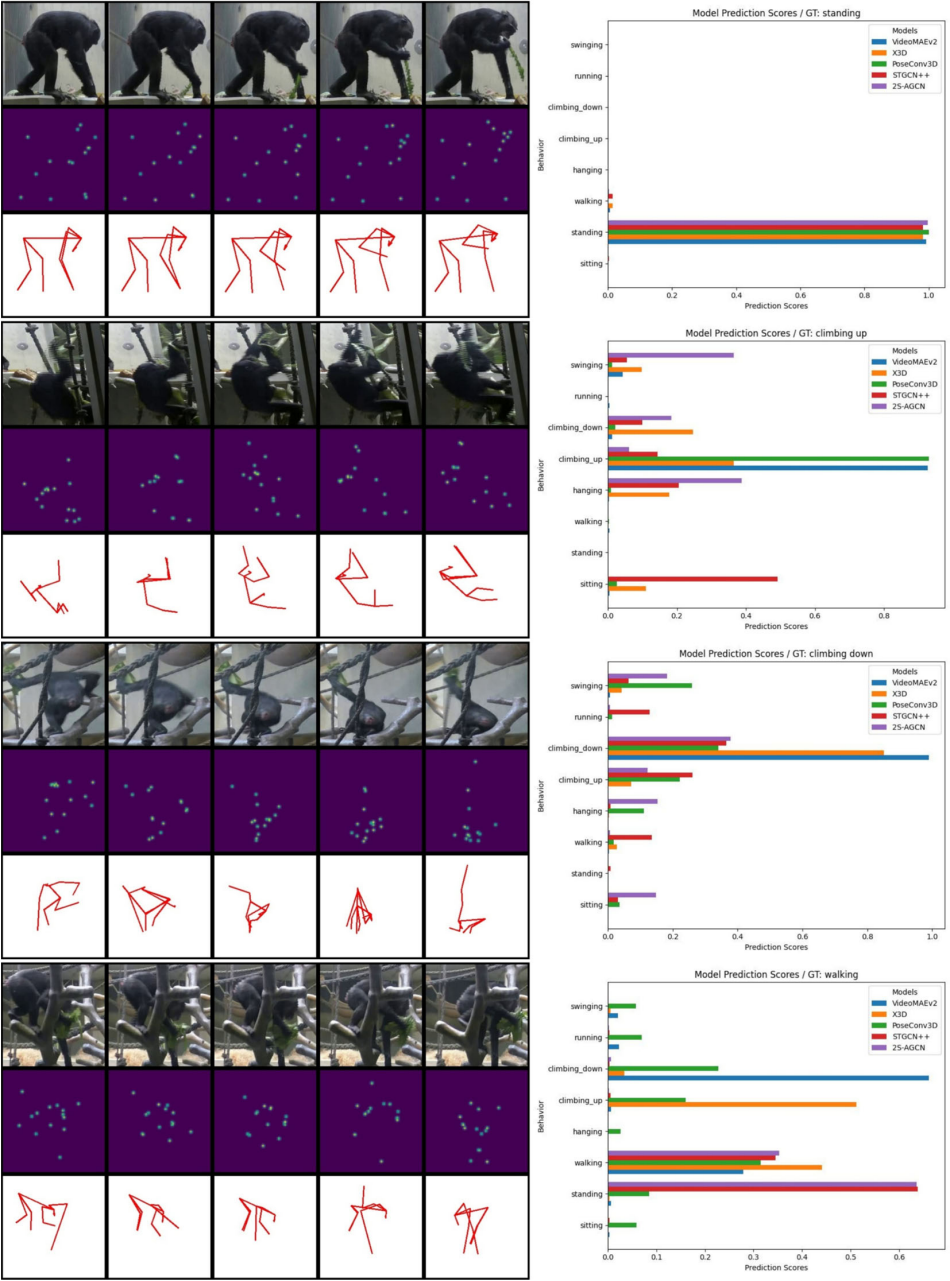
Examples of miniclips (left) and corresponding model prediction scores (right). Each miniclip includes original RGB images (top), stacked heatmaps (middle), and estimated skeletons (bottom), serving as inputs for video-based models (VideoMAEv2, X3D), the CNN-skeleton-based model (PoseConv3D), and GCN-skeleton-based models (STGCN$++$, 2 S-AGCN), respectively. For clarity, only 5 images per miniclip are shown. While predictions are unanimous when individuals are clearly visible (e.g., first example), noticeable differences arise with occlusions or complex poses

**Overall Observations:** The average performance metrics and their corresponding 95% confidence intervals (CI) for all models on the ChimpBehave dataset with all eight behavioral classes are presented in Table 3, while examples of model prediction scores are shown in Fig. 8 (see also Fig. A12 in the Supplementary Materials for examples of UMAP visualizations).

As a general remark, we observe relatively high Top-1 Accuracy and weighted F1 scores for all models, while significantly lower results are recorded for MCA and mAP. Unlike Top-1 Accuracy and weighted F1, MCA and mAP assign equal importance to each behavioral class in the dataset. The lower scores for these metrics indicate that while models can predict behaviors well overall, some behaviors remain more challenging to recognize.

Several factors may contribute to this discrepancy. One major factor is the unbalanced distribution of miniclips across behavioral classes. As highlighted in Section 3, ChimpBehave exhibits a typical long-tailed class distribution, with head classes (’sitting,’ ‘standing,’ and ‘walking’) being overrepresented compared to tail classes (’hanging,’ ‘climbing up,’ ‘climbing down,’ ‘running,’ and ‘swinging’).

When examining each model individually, we note that VideoMAEv2, the large video foundation model, achieves higher mean scores than any other model across all four evaluation metrics. The performance of X3D, the other video-based model, ranks second across all metrics. The three skeleton-based models exhibit very similar mean scores, all of which are inferior to those of the video-based approaches.

Regarding the statistical significance of the models’ ranking, the confidence intervals in Table 3 reveals the following:

(i) VideoMAEv2 is statistically better than X3D for only one performance metric, MCA, while the differences for the other three metrics are not statistically significant.
(ii) In contrast, VideoMAEv2 performs statistically better than all three skeleton-based models across all four metrics.
(iii) The higher performance of X3D compared to the three skeleton-based models is statistically significant only for MCA and mAP, the two metrics that assign equal importance to all classes.

**Class-Level Observations:** To gain better insights into the models’ ability to recognize different behaviors, we report class-level metrics in Table A1 and visualize the corresponding confusion matrices in Fig. A11 in the Supplementary Materials.

We observe that Precision, Recall, and F1 Score tend to be higher for the head classes (’sitting,’ ‘standing,’ and ‘walking’) compared to the less-frequent tail classes across all models. Additionally, the high false positive rate (FPR) for ‘sitting’ in all models highlights their tendency to overfit this class, likely due to its high frequency in the dataset. This overfitting is particularly pronounced when model training and selection are based on the Top-1 Accuracy score, as in our current setup.

Several factors may contribute to the difficulty in recognizing certain behaviors. For instance, behavior duration can vary significantly: individuals may sit for extended periods, whereas running is typically brief, making short-duration behaviors harder to classify. Moreover, handheld video recordings introduce variability in video quality, further complicating the recognition of fast-moving behaviors. Finally, some behaviors, such as sitting or walking, are visually more uniform across scenes, while others, like climbing down, can appear vastly different depending on the environment (e.g., descending a rope versus a platform).

When examining the video-based models, VideoMAEv2 and X3D, it becomes clear that ‘swinging’ is particularly difficult to classify, with mean F1 Scores of 38.4% and 25%, respectively. The confusion matrices reveal that these models often misclassify ‘swinging’ as ‘hanging,’ likely due to the significant visual similarity between these behaviors.

Skeleton-based models, on the other hand, show particularly low scores for fast-paced behaviors, such as ‘climbing down,’ ‘running,’ and ‘swinging,’ which often involve more complex poses. This reduced performance may result from inaccurate or noisy pose estimations for these behaviors. Notably, the underlying pose estimation model is trained on public datasets, which may be biased towards more common ape poses, making it less effective for these challenging behaviors.

**Table 4:** Within-dataset performance metrics and 95% confidence intervals (CI) for all models on ZOO and FOREST

		Model	Mode	Type	Pretraining	Top1	F1	MCA	mAP
ZOO	mean	VideoMAEv2	VB	ViT	UH/K400	**94**.**0**	**93**.**9**	**80**.**2**	**88**.**1**
		X3D	VB	CNN	K400	90.3	90.3	67.2	74.5
		PoseConv3D	SB	CNN	FineGym	87.9	87.6	55.7	63.7
		STGCN$++$	SB	GCN	NTU60	88.6	88.2	57.5	63.6
		2 S-AGCN	SB	GCN	NTU60	88.4	87.7	55.4	62.8
	CI	VideoMAEv2	VB	ViT	UH/K400	[92.1, 95.9]	[92.0, 95.7]	**[78.3, 82.2]**	[84.0, 92.2]
		X3D	VB	CNN	K400	[87.2, 93.5]	[87.2, 93.3]	[57.3, 77.1]	[63.0, 86.0]
		PoseConv3D	SB	CNN	FineGym	[85.8, 89.9]	[85.9, 89.2]	[52.4, 59.1]	[60.8, 66.6]
		STGCN$++$	SB	GCN	NTU60	[86.6, 90.6]	[86.8, 89.6]	[53.5, 61.6]	[59.1, 68.1]
		2 S-AGCN	SB	GCN	NTU60	[86.4, 90.5]	[85.7, 89.7]	[49.2, 61.6]	[55.9, 69.8]
FOREST	mean	VideoMAEv2	VB	ViT	UH/K400	**85**.**2**	**84**.**9**	**68**.**9**	**76**.**5**
		X3D	VB	CNN	K400	81.4	81.0	55.7	64.3
		PoseConv3D	SB	CNN	FineGym	73.8	72.8	46.7	55.6
		STGCN$++$	SB	GCN	NTU60	72.8	71.7	44.0	54.7
		2 S-AGCN	SB	GCN	NTU60	72.6	71.2	46.5	54.0
	CI	VideoMAEv2	VB	ViT	UH/K400	[81.2, 89.1]	[80.4, 89.4]	[58.8, 79.0]	[69.6, 83.4]
		X3D	VB	CNN	K400	[77.5, 85.2]	[77.1, 85.0]	[52.2, 59.3]	[58.7, 69.9]
		PoseConv3D	SB	CNN	FineGym	[71.4, 76.3]	[70.3, 75.4]	[43.7, 49.6]	[48.4, 62.7]
		STGCN$++$	SB	GCN	NTU60	[70.0, 75.6]	[68.6, 74.8]	[39.3, 48.7]	[49.3, 60.0]
		2 S-AGCN	SB	GCN	NTU60	[70.3, 74.9]	[69.0, 73.4]	[38.2, 54.9]	[46.4, 61.6]

### Within-Dataset Behavior Recognition

For the following analyses, we restrict the set of behavioral classes to the seven common classes present in both ChimpBehave and PanAf datasets: ‘sitting,’ ‘standing,’ ‘walking,’ ‘hanging,’ ‘climbing up,’ ‘climbing down,’ and ‘running.’ To emphasize the unique visual features of each dataset and simplify discussion, we refer to these subsets as ZOO and FOREST in the following sections.

**Overall Observations:** The performance metrics and confidence intervals of all models for the scenario within-dataset are shown in Table 4. Similar to the findings in Sect. 5.1, we observe a discrepancy between the generally high Top-1 Accuracy and F1 Score and the relatively lower MCA and mAP values. The former two metrics emphasize individual miniclips, while the latter two average performance across classes, giving each class equal weight. As previously discussed, this discrepancy can be attributed to unbalanced class distributions and of certain behaviors being inherently more difficult to distinguish. By detailing the observations to each specific dataset, we may note the following:

**ZOO**

(i) Similar to the results underlined in Sect. 5.1, VideoMAEv2 achieves the highest overall mean scores across all metrics, performs statistically better than X3D only for MCA, and outperforms statistically significantly all three skeleton-based methods across all metrics.
(ii) X3D achieves the second-highest mean scores across all metrics; however, these differences are not statistically significant when compared to the skeleton-based methods.
(iii) All skeleton-based methods performances are statistically tied.

**FOREST**

(i) VideoMAEv2 continues to exhibit the highest mean scores across all metrics, though none is statistically significant compared to X3D.
(ii) Similarly as in ZOO, VideoMAEv2 performs statistically better than all skeleton-based methods across all metrics in FOREST.
(iii) X3D achieves the second-highest mean scores, statistically better for Top-1 Accuracy and F1 Score compared to all three skeleton-based models.
(iv) The performances of all skeleton-based models are statistically tied.

**Dataset Comparison:**

(i) When comparing performance between ZOO and FOREST, all models achieve statistically significantly better results in ZOO for Top-1 Accuracy and F1 Score. Three major visual factors likely contribute to this result: (a) higher contrast between individuals and their backgrounds in ZOO, as shown in Fig. A10 (Supplementary Materials), (b) higher image resolution in ZOO (1920x1080) compared to FOREST (720x404), and (c) smaller relative size of individuals in FOREST, as the camera is fixed, whereas focal sampling in ZOO tracks each individual more closely.
(ii) For MCA and mAP, a different pattern emerges: for almost all models, the positive score difference between ZOO and FOREST is not statistically significant. Exceptions include MCA for PoseConv3D and ST-GCN$++$ and mAP for VideoMAEv2, which achieve statistical significance.

**Table 5:** Cross-dataset performance metrics and 95% confidence intervals (CI) for all models on ZOO$\rightarrow$FOREST and FOREST$\rightarrow$ZOO

		Model	Mode	Type	Pretraining	Top1	F1	MCA	mAP
ZOO$\rightarrow$FOREST	mean	VideoMAEv2	VB	ViT	UH/K400	**60**.**4**	**57**.**1**	**49**.**4**	**49**.**7**
		X3D	VB	CNN	K400	49.0	40.7	24.4	29.1
		PoseConv3D	SB	CNN	FineGym	58.1	54.7	35.4	37.2
		STGCN$++$	SB	GCN	NTU60	55.8	52.9	33.4	33.8
		2 S-AGCN	SB	GCN	NTU60	55.3	51.4	31.0	34.9
	CI	VideoMAEv2	VB	ViT	UH/K400	[56.6, 64.1]	[52.2, 62.0]	[44.5, 54.3]	[46.5, 53.0]
		X3D	VB	CNN	K400	[47.5, 50.5]	[36.9, 44.5]	[21.0, 27.8]	[27.6, 30.6]
		PoseConv3D	SB	CNN	FineGym	[56.4, 59.9]	[52.6, 56.8]	[33.0, 37.8]	[35.3, 39.0]
		STGCN$++$	SB	GCN	NTU60	[52.0, 59.6]	[49.1, 56.7]	[28.0, 38.8]	[30.5, 37.1]
		2 S-AGCN	SB	GCN	NTU60	[53.4, 57.2]	[47.3, 55.4]	[25.4, 36.5]	[33.6, 36.1]
FOREST$\rightarrow$ZOO	mean	VideoMAEv2	VB	ViT	UH/K400	74.6	77.6	**54**.**4**	**62**.**6**
		X3D	VB	CNN	K400	53.8	59.8	42.6	41.0
		PoseConv3D	SB	CNN	FineGym	78.6	79.8	47.2	46.2
		STGCN$++$	SB	GCN	NTU60	77.4	79.3	48.7	47.4
		2 S-AGCN	SB	GCN	NTU60	**79**.**3**	**80**.**6**	48.7	46.2
	CI	VideoMAEv2	VB	ViT	UH/K400	[70.8, 78.3]	[74.0, 81.2]	[52.9, 56.0]	[59.4, 65.9]
		X3D	VB	CNN	K400	[44.9, 62.7]	[51.1, 68.5]	[40.7, 44.4]	[37.7, 44.2]
		PoseConv3D	SB	CNN	FineGym	[75.8, 81.3]	[77.6, 82.1]	[44.3, 50.0]	[44.4, 48.0]
		STGCN$++$	SB	GCN	NTU60	[74.5, 80.3]	[77.5, 81.2]	[47.4, 50.1]	[44.5, 50.2]
		2 S-AGCN	SB	GCN	NTU60	[76.9, 81.6]	[79.1, 82.1]	[46.6, 50.9]	[43.2, 49.2]

### Evaluating Generalization: Cross-Dataset Results

The performance metrics of all models for cross-dataset setting (i.e., trained on one dataset and tested on the other) and their corresponding confidence intervals are provided in Table 5.

**Overall Observations:** In general, all models in the cross-dataset setting exhibit statistically significantly lower scores across all four metrics compared to their within-dataset performance, except for 2 S-AGCN’s MCA in the FOREST$\rightarrow$ZOO setting.

**From ZOO to FOREST:** When models are trained on ZOO and tested on FOREST, we observe the following:

(i) VideoMAEv2 still achieves the highest mean scores across all metrics; however, while the model is statistically significantly better than any model on MCA and mAP metrics, it is not statistically better than all skeleton-based models for Top-1 Accuracy and F1 scores.
(ii) X3D exhibits the worse performance with statistical significance across all metrics compared to all other models, except when compared to ST-GCN$++$’s mAP and 2 S-AGCN’s MCA.
(iii) The performances of all three skeleton-based models are statistically tied across all four metrics.

**From FOREST to ZOO:** All models achieve higher mean scores (often with statistical significance) when trained on FOREST and tested on ZOO compared to the opposite setting. This is expected, as the within-dataset task in ZOO showed overall higher mean scores than in FOREST. Models appear to perform better when trained in a challenging environment and tested in a easier one. Specific observations include:

(i) All three skeleton-based models achieve higher mean scores for Top-1 Accuracy and F1 Score compared to VideoMAEv2, although these differences are not statistically significant.
(ii) VideoMAEv2 outperforms all other models with statistical significance for MCA and mAP.
(iii) X3D performs statistically worse compared to all other models across all metrics, except when compared to PoseConv3D’s MCA and 2 S-AGCN’s mAP (not statistically significant).
(iv) The performances of all three skeleton-based models are statistically tied across all four metrics.

**Approach Comparison:** The comparatively higher robustness of the skeleton-based approaches compared to the video-based X3D aligns with previous findings in human-centric action recognition, where skeleton-based methods were shown to be less sensitive to visual changes, such as variations in appearance, background, and illumination (Han et al., 2017). This robustness is particularly relevant in the ZOO$\rightarrow$FOREST setting, as the FOREST dataset includes nighttime infrared recordings and captures the behaviors of two great ape species, chimpanzees and gorillas. However, recent large video-based foundation models, such as VideoMAEv2, are less affected by these visual changes and generalize well in cross-dataset settings.

**Class-Level Observations:** As shown in Table A2 in the Supplementary Materials, in the ZOO$\rightarrow$FOREST setting all models tend to predict many instances as ‘sitting,’ the predominant class in the training dataset. This is reflected by the extremely high average False Positive Rate (FPR) for ‘sitting’ (e.g., VideoMAEv2: $46.0\%$, X3D: $72.7\%$, PoseConv3D: $49.9\%$). Conversely, many test examples of the other two head classes, ‘standing’ and ‘walking,’ are missed, as indicated by their high average False Negative Rate (FNR) (e.g., for ‘standing’, VideoMAEv2: $67.7\%$, X3D: $86.5\%$, PoseConv3D: $69.1\%$). Consequently, the average F1 scores for these head classes are relatively low: e.g., for ‘sitting, the F1 score achieved by VideoMAEv2 is $74.2\%$ (respectively $64.5\%$ for X3D and $71.4\%$ for PoseConv3D), whereas for ‘standing,’, the F1 score achieved by VideoMAEv2 is $42.0\%$ (respectively $21.6\%$ for X3D nd $41.6\%$ for PoseConv3D).

In contrast, in the FOREST$\rightarrow$ZOO setting (see Table A3 in the Supplementary Materials), head classes are recognized much better overall, as evidenced by their average F1 scores: e.g., for ‘sitting,’ VideoMAEv2 achieves an F1 score of $86.3\%$ (respectively $67.4\%$ for X3D and $91.5\%$ for PoseConv3D), whereas for ‘standing, VideoMAEv2 achieves an F1 score of $61.5\%$ (respectively $43.1\%$ for X3D nd $67.1\%$ for PoseConv3D). This may partially explain why the performance decline is more pronounced in ZOO$\rightarrow$FOREST than in FOREST$\rightarrow$ZOO.

## Discussion

In this section, we discuss the results of our experiments, highlight some limitations - especially in the context of real-life applications - and make suggestions for future research.

The ChimpBehave dataset, introduced in Sect. 3, is currently one of the largest great ape-specific datasets for behavior recognition publicly available. Unlike previous datasets, its annotations are curated by an expert primatologist, ensuring high-quality and contextually accurate labeling. Additionally, the dataset features significantly higher video resolution compared to existing resources. ChimpBehave also provides a novel platform for researchers to investigate methods in domain adaptation and out-of-distribution generalization, which could lead to the development of animal behavior recognition models that are more robust to visual and contextual variability.

However, this dataset has several limitations, the most significant being its unbalanced class distribution. While certain behaviors, like sitting and walking, naturally occur more frequently than others, such as hanging or running, their dominance in the data distribution may affect overall model performance, resulting in a tendency to overfit to the more prevalent classes. In the case of ChimpBehave, data augmentation techniques could help mitigate this class frequency disparity, such as oversampling underrepresented classes or creating miniclips with overlapping video frames. Another approach could involve implementing weighted loss functions that incentivize correct classification of less frequent behaviors. From a practical perspective, in real-life scenarios - such as an automated system monitoring animal health or well-being - one would want the system to reliably detect behaviors like swinging. However, in our experiments, models often confuse swinging with hanging, which would be unsuitable for such applications. Future research should place special emphasis on behaviors that, while less frequently displayed, convey high ecological importance. In addition, compared to other datasets that emphasize annotations of social interactions among conspecifics in great apes [e.g. ChimpACT (Ma et al., 2023)], our dataset focuses exclusively on locomotive behaviors of single individuals, which may limit its applicability to certain aspects of primatology.

Our experiments were designed to evaluate and compare model performance both within visually distinct datasets and in cross-dataset settings, with the goal of assessing their capacity for out-of-distribution generalization. This study focuses on a variety of model architectures, including VideoMAEv2 (Wang et al., 2023), a large video foundation model that achieves state-of-the-art (SOTA) performance in human-centric benchmarks; X3D (Feichtenhofer, 2020), a video-based SOTA 3D-CNN model; and three skeleton-based approaches: PoseConv3D (Duan et al., 2022), STGCN$++$ (Duan et al., 2022), and 2 S-AGCN (Shi et al., 2019), which leverage CNNs or GCNs applied to pose-estimated data. Using a strict 5-fold cross-validation procedure throughout our experiments, we ensured that model performance comparisons were statistically robust.

In summary, among all models evaluated across five dataset settings and four distinct metrics (20 evaluations per model), VideoMAEv2 achieved the highest mean scores in 18 of these settings, six of which reached statistical significance (see Tables 3 to 5). The remaining two highest evaluation scores were obtained by 2 S-AGCN, a skeleton-based approach. When excluding VideoMAEv2, X3D ranked highest in all 12 within-dataset evaluations (three with statistical significance) but performed the worst in all eight cross-dataset evaluations (four with statistical significance) compared to the three skeleton-based models. Among the skeleton-based approaches, performance was largely similar across all evaluations, with no statistically significant differences.

These results suggest that, within a specific dataset, video-based models generally outperform skeleton-based ones, particularly large transformers like VideoMAEv2. This model also demonstrated relative robustness to visual changes in cross-dataset settings, unlike more traditional video-based CNN architectures such as X3D, which exhibited a significant performance decline. In contrast, while skeleton-based models achieved lower average scores within datasets, they remained relatively robust to visual changes in cross-dataset settings, sometimes even surpassing VideoMAEv2.

At first glance, one might conclude from these findings that video foundation models such as VideoMAEv2, with their strong overall performance and the absence of a need for pose estimation during preprocessing, render skeleton-based approaches unnecessary. However, we offer two considerations in support of skeleton-based methods.

First, as noted in Sect. 5, the upstream pose estimation model used for the skeleton-based approaches was trained only on ChimpACT (Ma et al., 2023) and OpenApePose (Desai et al., 2022), two public datasets whose annotations are likely biased toward common ape poses associated with frequent behaviors, such as sitting or walking. As discussed in the class-level observations, this bias could explain why skeleton-based methods struggle with recognizing fast-paced behaviors involving complex poses, such as climbing down, running, or swinging. We believe that access to additional pose estimation datasets, annotated specifically for complex poses, could significantly improve the performance of skeleton-based methods in this study.

Second, for primatologists working in the field with limited resources, it is important to consider not only model efficacy but also computational efficiency. For example, training VideoMAEv2 on ZOO for a single epoch takes approximately one hour, whereas training STGCN$++$ under the same conditions takes only about 10 s (excluding the preprocessing time for pose estimation). Furthermore, VideoMAEv2 requires approximately 90 times more floating-point operations per second (FLOPs) than STGCN$++$ at inference time. This substantial computational demand makes VideoMAEv2 impractical for certain real-time applications or deployment on low-power devices.

While the results in Sect. 5 provide comparisons between video-based and skeleton-based behavior recognition models, they do not explore the potential of multimodal models that combine video and skeletal data. For instance, PoseConv3D, one of the skeleton-based models used in our experiments, natively supports such a design, and this approach has been shown to achieve higher performance on multiple human action datasets (Duan et al., 2022). Although we opted to evaluate each modality separately for research purposes, we believe that combining them could yield even higher performance. Furthermore, given the strong generalization performance of VideoMAEv2, scenarios where RGB videos are enriched with skeletal pose data-either displayed alone or superimposed onto the original video pixels-could lead to even stronger performance. Investigating these multimodal approaches represents an important avenue for future research.

When examining model generalization in cross-dataset settings (Sect. 5.3), our results reflect performance on out-of-distribution data without any fine-tuning on the target test data. In practice, researchers often have access to at least some labeled data from the target domain. Future experiments could explore scenarios where fine-tuning is applied to test whether the generalization capacity observed in our experiments remains robust. Similarly, as noted earlier, the pose estimation model used in our skeleton-based approaches was not fine-tuned on either the ChimpBehave or PanAf datasets. In practice, fine-tuning on these datasets is recommended, as the quality of estimated poses can significantly impact the final behavior classifier’s performance (Duan et al., 2022). Additionally, PanAf includes footage of both gorillas and chimpanzees, while ChimpBehave focuses exclusively on chimpanzees. Anatomical and behavioral differences between these species could pose challenges for algorithms trained solely on chimpanzee data. Future work could investigate within-PanAf performance differences between gorilla and chimpanzee footage, providing insights into species-specific model behavior and addressing the extent to which training exclusively on chimpanzees impacts cross-species generalization.

The field of animal behavior studies, like many other scientific domains, may be entering a new era where human effort is increasingly complemented by artificial intelligence. Technologies such as those presented in this paper could become valuable tools for researchers by, for example, pre-annotating regions of interest in video clips or discarding irrelevant sequences in lengthy footage. While frequently observed behaviors such as walking, sitting, and standing can often be accurately recognized by automated systems, less common behaviors involving more complex body movements - such as swinging - are still prone to misclassification. For many practical applications, this level of accuracy may not yet be sufficient for field deployment. However, studies on human actions have demonstrated the potential of machine learning models to accurately recognize highly sophisticated activities when larger datasets are available and pose estimations are more accurate. As more annotated behavioral data of non-human primates becomes accessible, we believe that automated systems will play an increasingly important role in efforts to halt biodiversity loss and protect endangered species more effectively.

## Data Availability

The ChimpBehave dataset generated and analyzed during the current study is available in the ChimpBehave repository, accessible at https://github.com/MitchFuchs/ChimpBehave. Additional datasets used, such as PanAf, OpenApePose and ChimpACT, can also be accessed as detailed in their respective publications (Brookes et al., 2024; Desai et al., 2022; Ma et al., 2023). Any further data requests can be directed to the corresponding author.
